# Supplementary material for: Leveraging large language models for rare disease named entity recognition
Source: PLOS Digit Health. 2026 Feb 12;5(2):e0001242. doi: 10.1371/journal.pdig.0001242 (PMC12900354; doi:10.1371/journal.pdig.0001242)
Supplement: S1 Fig — (DOCX) [file pdig.0001242.s001.docx]

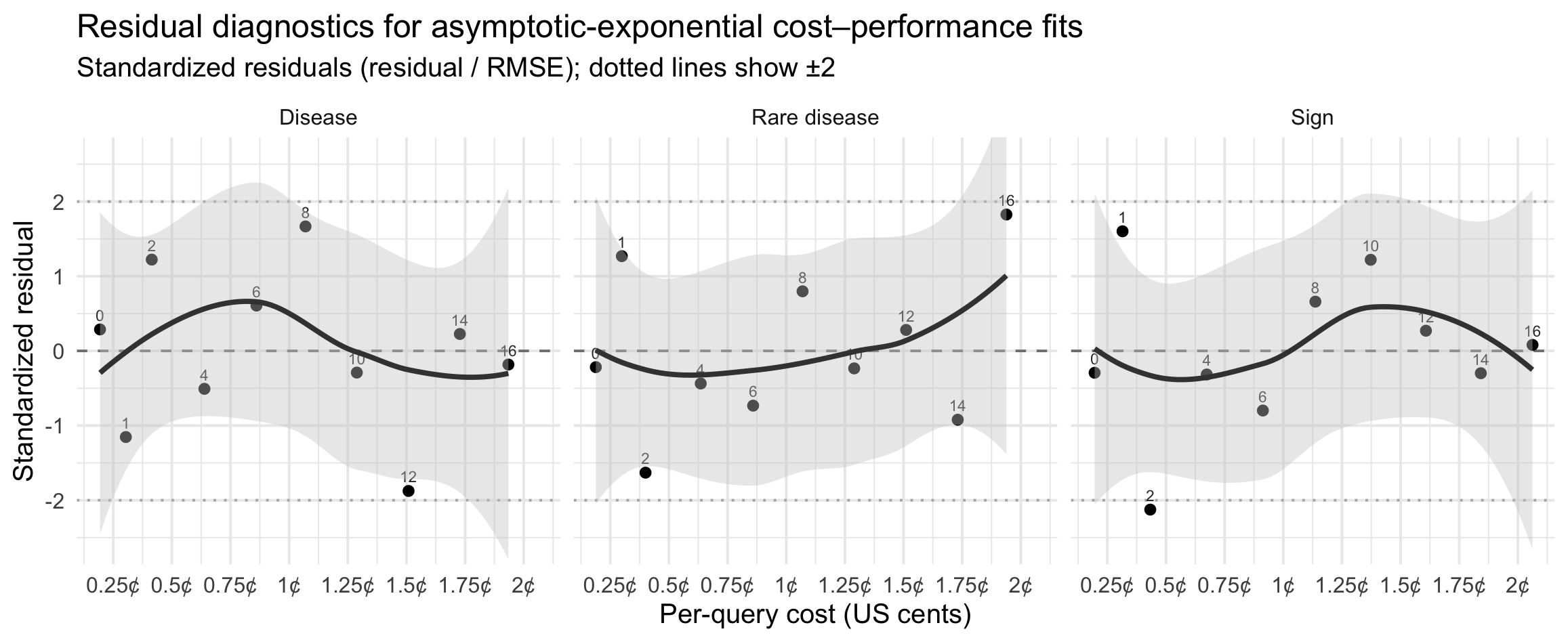


**S1 Fig. Residual diagnostics for asymptotic-exponential cost-performance models.** Each panel shows residuals $\left( \text{observed F1} - \text{fitted F1} \right)$ plotted against per-query cost for rare disease, disease, and sign. The dashed horizontal line indicates zero residual. Symptom is excluded because its curve is fitted with nonparametric LOESS.
