# Supplementary material for: Leveraging large language models for rare disease named entity recognition
Source: PLOS Digit Health. 2026 Feb 12;5(2):e0001242. doi: 10.1371/journal.pdig.0001242 (PMC12900354; doi:10.1371/journal.pdig.0001242)
Supplement: S1 Table — (DOCX) [file pdig.0001242.s002.docx]

**S1 Table. Paired document-level bootstrap tests for RAG effects.** For each entity type, we report paired differences Δ = (RAG − baseline) in precision, recall, and F1 for two prespecified comparisons: (i) zero-shot + RAG vs zero-shot and (ii) best performing few-shot setting + RAG vs its matched few-shot baseline. Values are bootstrap mean Δ with 95% confidence intervals in brackets. One-sided p-values less than 0.1 are underscored.

| **Entity** | **Comparison** | **Δ Precision** | **p** | **Δ Recall** | **p** | **Δ F1** | **p** |
| --- | --- | --- | --- | --- | --- | --- | --- |
| Rare Disease | Zero-shot + 2-RAG vs zero-shot | -0.041  (-0.104, 0.026) | 0.896 | -0.121  (-0.168, -0.075) | 1.000 | -0.108  (-0.152, -0.064) | 1.000 |
|  | 4-shot + 2-RAG vs 4-shot | 0.030  (-0.012, 0.074) | **0.076** | -0.058  (-0.095, -0.026) | 0.999 | -0.026  (-0.059, 0.002) | 0.965 |
| Disease | Zero-shot + 1-RAG vs zero-shot | -0.060  (-0.141, 0.022) | 0.927 | -0.022  (-0.074, 0.028) | 0.819 | -0.032  (-0.095, 0.028) | 0.850 |
|  | 4-shot + 1-RAG vs 4-shot | 0.025  (-0.035, 0.087) | 0.214 | -0.035  (-0.086, 0.018) | 0.916 | -0.011  (-0.059, 0.038) | 0.691 |
| Sign | Zero-shot + 1-RAG vs zero-shot | 0.029  (-0.009, 0.066) | **0.067** | 0.028  (0.006, 0.051) | **0.009** | 0.030  (0.005, 0.055) | **0.010** |
|  | 4-shot + 1-RAG vs 4-shot | -0.053  (-0.096, -0.011) | 0.993 | -0.097  (-0.143, -0.051) | 1.000 | -0.076  (-0.116, -0.037) | 1.000 |
| Symptom | Zero-shot + 1-RAG vs zero-shot | -0.032  (-0.062, -0.005) | 0.992 | 0.059  (0.000, 0.148) | 0.134 | -0.041  (-0.083, -0.001) | 0.976 |
|  | 4-shot + 1-RAG vs 4-shot | 0.002  (-0.013, 0.019) | 0.448 | 0.038  (0.000, 0.121) | 0.377 | 0.004  (-0.018, 0.032) | 0.403 |
