# Supplementary material for: Leveraging large language models for rare disease named entity recognition
Source: PLOS Digit Health. 2026 Feb 12;5(2):e0001242. doi: 10.1371/journal.pdig.0001242 (PMC12900354; doi:10.1371/journal.pdig.0001242)
Supplement: S2 Table — (DOCX) [file pdig.0001242.s003.docx]

**S2 Table. Goodness-of-fit diagnostics for the asymptotic-exponential cost-performance models.** Symptom is excluded because its curve is fit with nonparametric LOESS.

| Entity | n | RMSE | Pseudo $R^{2}$ |
| --- | --- | --- | --- |
| Rare disease | 10 | 0.0058 | 0.9068 |
| Disease | 10 | 0.0171 | 0.9162 |
| Sign | 10 | 0.0052 | 0.9909 |
